# Measurement of pulmonary arterial pulse wave reflection from single-slice phase-contrast and steady-state free precession MRI

**DOI:** 10.1186/1532-429X-14-S1-W35

**Published:** 2012-02-01

**Authors:** Peter Leimbigler, Joshua van Amerom, Lars Grosse-Wortmann, Shi-Joon Yoo, Chris Macgowan

**Affiliations:** 1 Medical Biophysics & Medical Imaging, University of Toronto & Hospital for Sick Children, Toronto, ON, Canada; 2 Diagnostic Imaging, Hospital for Sick Children, Toronto, ON, Canada; 3 Labatt Family Heart Centre, Toronto, ON, Canada

## Summary

Pulmonary arterial hypertension (PAH) is associated with elevated pulmonary vascular resistance, resulting in increased reflection of pressure and flow waves from distal vessels^1^. The gold standard for assessing PAH is right heart catheterization, an invasive procedure that carries a 5% risk of major complications^2^. We validate a noninvasive method for quantifying pulmonary arterial reflection using phase-contrast (PC) and steady-state free precession (SSFP) sequences acquired in a single slice.

## Background

**Figure 1:**
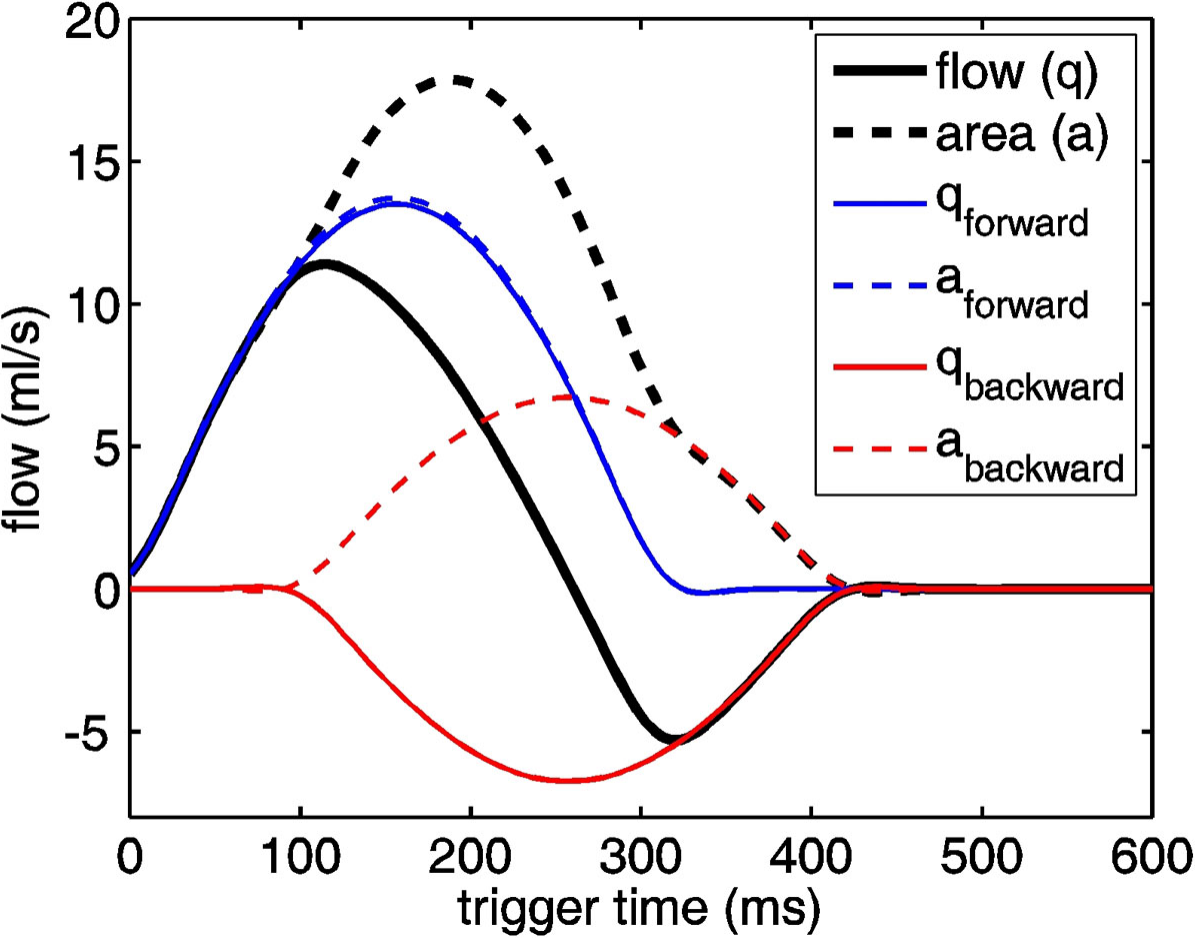
Illustration of single-slice wave separation. Before the onset of backward waves, flow and area are related by the constant factor *PWV*. Backward-traveling flow and area waves are equal in form but opposite in sign, and are proportional to the difference between *PWV* × area and flow.

An arterial segment approximates a hydraulic transmission line terminated distally by a reflection site that partially reflects forward-traveling pressure and flow (*q*) waves back toward the heart^3^. Due to finite pulse wave velocity (*PWV*), backward-traveling waves are minimal in early systole (Figure 1); since arterial cross-sectional area (*a*) increases roughly linearly with pressure, *PWV* = *∂q*(*t*)/*∂a*(*t*). Combining this with the water hammer equation yields an expression for the backward flow wave^4^:

<center>*q*_b_(*t*) = [*q*_meas_(*t*) - *PWV*×*a*(*t*)]/2,</center>from which arterial reflection magnitude (*R*) can be computed in the frequency domain:

<center>*R*(*ω*) = *Q*_b_(*ω*)/*Q*_f_(*ω*).</center>

## Methods

The right pulmonary artery in three healthy adult volunteers was imaged on a 1.5 T MR system (Siemens, Germany) using retrospectively ECG-gated cine PC and SSFP sequences to quantify blood velocity and vessel cross-section, respectively. PC and SSFP images were co-registered in MATLAB (The MathWorks, USA). The arterial lumen was outlined semi-automatically using Segment (Medviso, Sweden), yielding flow and area time series that were resolved into forward and backward flow waves in MATLAB. The frequency-domain ratios of backward to forward flow waves yielded estimates of *R* which were then averaged over the fundamental heart frequency and the next two harmonics^3^ and compared to literature values using a two-tailed Student's *t*-test.

## Results

**Figure 2:**
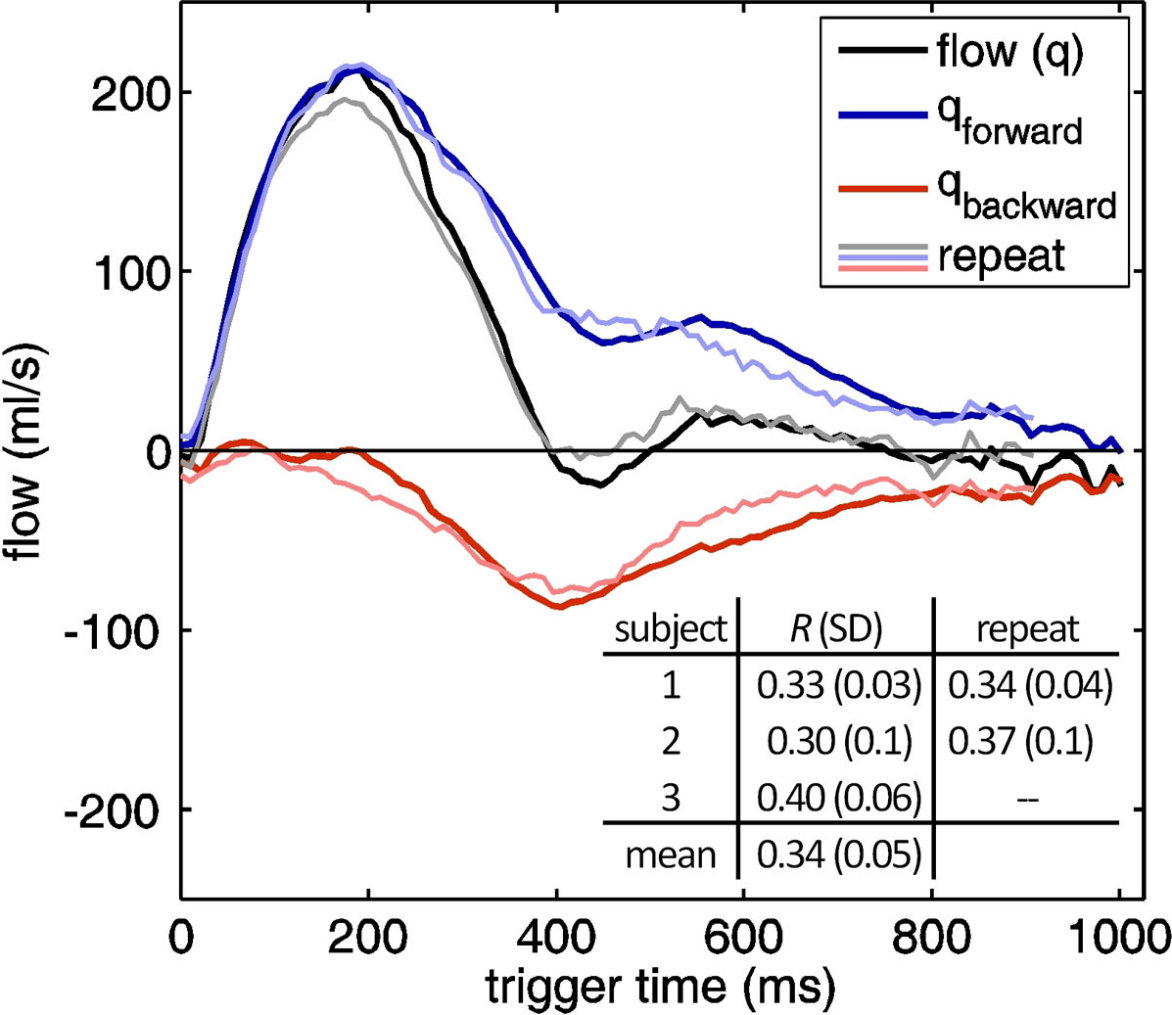
Flow wave separation in the right pulmonary artery of Subject 1 calculated from an initial MR study and a repeat scan 36 days later. Reflection magnitudes and standard deviations are tabulated for all subjects. Scan parameters were TR/TE = 9.8/2.2 ms, 192 × 192 matrix, 28 × 28 cm^2^ FOV, 2 averages, flip angle = 10° (PC) and 45° (SSFP). The root-mean-variances of the forward, measured and backward flow waves between scans were 11, 16 and 8 ml/s, respectively.

The single-slice MRI method reliably resolved forward and backward flows *in vivo* (Figure 2), enabling noninvasive measurement of normal right pulmonary arterial reflection magnitudes, *R* (SD) = 0.34 (0.05), statistically equivalent (*p* = 0.74) to invasively measured literature values^5^ of *R* (SD) = 0.33 (0.13).

## Conclusions

The feasibility of single-slice MRI measurement of pulmonary arterial reflection in healthy adults motivates follow-up studies in adult and pediatric patient populations and lays the groundwork for noninvasive assessment of pulmonary hypertension.

## Funding

This study was supported by the Canadian Institutes of Health Research (App #199854).
